# Olanzapine-Induced Hyponatremia Presenting with Seizure Requiring Intensive Care Unit Admission

**DOI:** 10.7759/cureus.8212

**Published:** 2020-05-20

**Authors:** Michelle A McNally, Mehmet Camkurt, Lokesh R Shahani, Rania Elkhatib

**Affiliations:** 1 Dermatology, John P. and Kathrine G. McGovern School of Medicine, University of Texas Health Center, Houston, USA; 2 Psychiatry, John P. and Kathrine G. McGovern Medical School at University of Texas Health Science Center, Houston, USA

**Keywords:** hyponatremia, olanzapine, siadh, psychogenic polydipsia, seizure, atypical antipsychotics, schizophrenia, pharmacology

## Abstract

Rapid-onset hyponatremia is a rare, but potential, complication of olanzapine treatment. Hyponatremia, secondary to atypical antipsychotic use, has been reported in many case reports and is thought to be associated with a syndrome of inappropriate anti-diuretic hormone secretion (SIADH). Psychogenic polydipsia is a separate cause of hyponatremia, which is also seen in the psychiatric population, particularly in schizophrenia, and must be differentiated from SIADH. We report a case of sudden-onset hyponatremia resulting in seizure onset necessitating intensive care unit admission in a patient taking olanzapine during hospitalization in a psychiatric unit. Clinicians should be aware of the association between olanzapine and hyponatremia secondary to SIADH or psychogenic polydipsia. As in our case, the patient status may decline rapidly, resulting in seizure onset. Physicians should actively monitor patients taking antipsychotics for changes in serum sodium levels.

## Introduction

Hyponatremia is a relatively uncommon side effect of psychotropic medications in clinical practice [1]. When present, hyponatremia may be a strong indicator of the syndrome of inappropriate anti-diuretic hormone secretion (SIADH). Although hyponatremia may be asymptomatic, it may also present with symptoms such as nausea, fatigue, confusion, and disorientation [2]. Occasionally, it may result in severe neurological symptoms including coma, seizures, and death [2]. More severe side effects tend to develop in cases of prominent sodium decrease within a short period of time [3]. Hyponatremia as a result of SIADH can be secondary to central nervous system disorders, neoplasms, pulmonary diseases, and medications [4-5]. In psychiatric practice, hyponatremia secondary to SIADH is usually associated with selective serotonin reuptake inhibitors (SSRIs) and is more commonly seen in the elderly population [6]. With SSRI treatment, the median time of hyponatremia onset is 13 days with 57% of cases occurring within 30 days of medication initiation [3]. Although seen less frequently than with SSRI use, hyponatremia secondary to SIADH can also develop with antipsychotic use [7].

Psychogenic polydipsia is a phenomenon characterized by disordered water balance with excessive water intake and is another cause of hyponatremia that has been frequently described in the psychiatric population, primarily in schizophrenia, with prevalence ranging from 6.2% to 20% [8]. Although polydipsia alone is rarely sufficient to cause marked hyponatremia, unexplained concomitant defects in ADH secretion, urinary dilution, and the regulation of osmolality have also been found in psychotic patients and may contribute to the hyponatremia seen in psychogenic polydipsia [9]. Psychogenic polydipsia is a diagnosis of exclusion that must only be considered, once other organic causes of hyponatremia have been ruled out [10].

We report a case of rapid onset of hyponatremia in a patient taking olanzapine, which presented with a seizure and required intensive care unit admission.

## Case presentation

The reported patient is a 34-year-old male with a past psychiatric history of schizoaffective disorder, who presented to the medical emergency department after experiencing aggressive behavior and voicing suicidal ideations. He had been prescribed antipsychotics in the past but denied any current medication use. Upon initial assessment, the patient was pacing, screaming, self-talking, and responding to internal stimuli. Point-of-care labs were drawn, which revealed a low serum sodium level of 131 mEq/L and a low chloride level of 94 mEq/L. Urine drug screen was negative. Psychiatry was consulted, the patient was started on olanzapine (10 mg/day), and it was recommended that the patient be transferred to an inpatient psychiatric facility for further management.

Upon arrival at the psychiatric facility, labs showed a serum sodium concentration of 142 mEq/L. Olanzapine was increased to 20 mg/day to target his psychotic symptoms. While at the facility, the patient was noted to be drinking water, but no excessive water intake was reported by the nursing staff or observed by the treatment team. On the fifth day of his stay, the patient experienced a witnessed, tonic-clonic seizure that lasted for about one minute. Post seizure, the patient’s mental status was altered, and he was transferred to the emergency department at a nearby hospital for medical evaluation. 

After arrival at the emergency department, the patient experienced two episodes of emesis. The workup revealed an acute drop in serum sodium to 119 mEq/L. A significant point-of-care basic metabolic panel labs showed a low serum osmolality (249 mOsmol/kg H_2_O) with a normal chloride level (82 mEq/L). Urinalysis showed urine sodium level of 21 mEq/L, urine potassium level of 6 mEq/L, urine chloride level of 10 mEq/L, low specific gravity (1.0003), and urine osmolality of 93 mOsm/kg H_2_O.

The patient was transferred to the intensive care unit for further evaluation and treatment. On assessment, the patient was thin, tachycardic, and afebrile. He was unable to respond to his name and open his eyes. He was positive for upper extremity rigidity, restlessness, attempting to get out of bed, and pulling on his nasogastric tube. The patient was given fosphenytoin, 20 mg/kg. The hyponatremia was treated with 3% saline (50 mL x 4 doses overnight) and the hyponatremia and altered mental status resolved with a repeat sodium level of 137 mEq/L the following day.

## Discussion

A recent literature review of case reports searched from 1946 through 2016 concluded that hyponatremia is a potential complication of treatment with second-generation antipsychotics in patients diagnosed with schizophrenia [11]. Another study searched the WHO database for safety reports of adverse drug reactions using VigiBase and found an association between antipsychotic use and hyponatremia, with olanzapine (n=163) being the second most reported atypical antipsychotic to be associated with hyponatremic events [12]. Anil *et al*. reported a case of olanzapine-induced sudden-onset hyponatremia, on the second day of initiation, in a patient with a 15-year history of bipolar affective disorder who had been off of medication for the last 7 years [13]. The patient was found with altered mental status; her sodium level was 128 mEq/L. Based on these findings, olanzapine was withdrawn, and the hyponatremia subsequently resolved over the following four days [13].

Urine studies can aid in diagnosing the cause of hyponatremia in patients taking antipsychotics who present with hyponatremia (serum sodium level <135 mEq/L). Patients can be classified as having SIADH and differentiated from those with polydipsia by urine sodium levels and urine osmolality. Those presenting with urine sodium >20 mEq/L and urine osmolality >100 mOsmkg are more likely to have SIADH, while those with urine sodium <20mEq/L and urine osmolality <100mOsm/kg are more likely to have polydipsia [14]. However, diagnosis may not be clear in every case. Riggs *et al*. suggest that the pathology of water homeostasis in psychiatric patients may be multifactorial and may be explained by compulsive water ingestion as seen in polydipsia, SIADH induced, or by a combination of the aforementioned factors [15]. In addition, observation of the amount of water intake by the patient requires ongoing monitoring and staff resources. For these reasons, distinguishing between psychogenic polydipsia and antipsychotic-induced hyponatremia may be complicated, as highlighted by our case.

Our patient's severe presentation with altered mental status and seizure activity, secondary to acute-onset hyponatremia, was most likely complicated by olanzapine use. The upper-extremity rigidity, restlessness, and agitation may also be explained by olanzapine use, but the fact that the patient’s mental status improved following administration of hypertonic saline makes hyponatremia the more likely culprit.

Although our patient was not noted to have excessive water intake, it is possible that he may have been drinking excessively while in the restroom. In addition, a urine osmolality of 93 mOsmkg (< 100 mOsmkg) favors the diagnosis of psychogenic polydipsia. However, most patients with psychogenic polydipsia remain normonatremic [16]. In regards to the urine sodium, a level of 21 mEq/L (>20mEq/L) suggests SIADH. In line with Riggs *et al.*, our patient's presentation suggests that the cause of the hyponatremia may be multifactorial in nature.

Before making a diagnosis of SIADH, other causes of elevated ADH should be excluded. Heart failure, cirrhosis, dehydration, thyroid, and adrenal dysfunction are all potential causes of elevated ADH that must first be ruled out before making a diagnosis of SIADH. In our case, the patient was not worked up for heart, liver, thyroid, or adrenal disease, which further complicates his diagnosis.

The mechanism for the development of SIADH/hyponatremia secondary to antipsychotic use is unknown. However, it is postulated that antipsychotics may act as a stimulus for ADH release, enhance ADH activity in the kidneys, and may cause dopamine receptor supersensitivity from long-term blockade, elevating ADH levels [17]. It is also suggested that antipsychotics induce hyponatremia through polydipsia by causing dry mouth or stimulating the thirst center in the brain [12]. Interestingly, schizophrenia has been considered to be associated with hyponatremia per se [18]. Some patients with a diagnosis of schizophrenia are considered to have a condition called “the syndrome of psychosis, intermittent hyponatremia and polydipsia” [19]. This subtype of patients with schizophrenia who are chronically hyponatremic and polydipsic demonstrates increased ADH secretion, which may be due to hippocampal dysfunction [9]. Our patient was noted to have a low sodium level of 131 mEq/L at the onset of hospitalization, which might be the initial explanation of his imbalance in sodium homeostasis. Given the fact that our patient initially presented with a low sodium level that was further complicated by seizure activity along with his concurrent olanzapine use makes differential diagnosis more challenging.

Treatment of hyponatremia in the psychiatric population should begin with primary prevention. Information regarding the patient’s water intake, both before the initiation of antipsychotics and during treatment, should be assessed [14]. The patient should be educated on avoiding excessive water ingestion and monitored appropriately. Serum sodium levels should be checked periodically during treatment, as well after dosage adjustments. For asymptomatic patients with hyponatremia, water restriction should be considered initially [14]. Acute, severe hyponatremia, as seen in our patient, presenting with altered mental status and neurologic sequelae requires urgent treatment. Administration of 3% hypertonic saline should be performed to correct hyponatremia. The initial rate of sodium correction should not exceed 1 to 2 mmol per L per hour, and sodium levels should be monitored closely, to prevent central pontine myelinolysis, a devastating complication of rapid, overzealous correction of sodium levels [20].

## Conclusions

In summary, this case is important in raising awareness of the possible complication of rapid onset hyponatremia, which may occur in psychiatric patients taking olanzapine. As in our case, patient status may decline rapidly, resulting in seizure onset. In cases such as these, it is important to take into account both possibilities of SIADH and psychogenic polydipsia as contributors to hyponatremia. It is also important to recognize that a subset of schizophrenia patients might have baseline hyponatremia with polydipsia and may be more susceptible to hyponatremia when taking olanzapine or other antipsychotic drugs. Differential diagnosis of SIADH and psychogenic polydipsia can be challenging. Physicians should actively monitor patients taking antipsychotics periodically for changes in serum sodium levels.
